# Effect of dotinurad versus febuxostat on the one-year eGFR slope in CKD patients with hyperuricemia: a retrospective cohort study

**DOI:** 10.1186/s12882-026-04937-7

**Published:** 2026-04-10

**Authors:** Hikaru Uematsu, Tatsunori Toida, Yuri Tanaka, Nobuhiko Joki

**Affiliations:** Division of Nephrology, Toho University Ohashi Medical Center, 2-22-36 Ohashi, Meguro-ku, Tokyo, 153-8515 Japan

**Keywords:** Dotinurad, Febuxostat, Chronic kidney disease, Hyperuricemia, eGFR slope, Urate-lowering therapy, Selective urate reabsorption inhibitor

## Abstract

**Background:**

The renoprotective effects of urate-lowering therapy, especially with selective urate reabsorption inhibitors (SURIs), remain to be established in patients with chronic kidney disease (CKD). This study compared changes in the estimated glomerular filtration rate (eGFR) slope associated with the initiation of dotinurad (a SURI) versus febuxostat (a xanthine oxidase inhibitor) in patients with chronic kidney disease, mainly due to diabetic kidney disease or hypertensive nephrosclerosis.

**Methods:**

We conducted a single-center retrospective cohort study of 81 adult CKD patients with hyperuricemia who were newly started on dotinurad (*n* = 31) or febuxostat (*n* = 50) between January 2020 and October 2023. The primary endpoint was the change in the annual eGFR slope in the 1-year period before and after therapy initiation, which was evaluated via a linear mixed-effects model adjusted for baseline differences and confounders.

**Results:**

According to the multivariable-adjusted analyses, the change in the eGFR slope differed significantly between the two groups (interaction term for group × period × time, *p* = 0.048). In the dotinurad group, a declining eGFR trend of − 2.8 mL/min/1.73 m² per year (95% confidence interval [CI] − 6.0 to + 0.4) before treatment stabilized to + 1.4 mL/min/1.73 m² per year (95% CI − 1.0 to + 3.7) after one year of therapy. This within-group change (+ 4.2 mL/min/1.73 m² per year) was statistically significant (*p* = 0.021). No comparable change was observed in the febuxostat group, whose eGFR slope changed from − 0.3 (95% CI − 3.0 to + 2.5) to − 0.8 (95% CI − 2.6 to + 1.0) mL/min/1.73 m² per year (within-group change − 0.5, *p* = 0.731). This difference in slopes accounted for the overall difference in treatment effects between the two therapies. The proportion of patients who achieved the target serum uric acid concentration < 6.0 mg/dL at 12 months did not differ significantly between the groups, nor did the incidence of a ≥ 30% decline in the eGFR during follow-up.

**Conclusions:**

In this CKD cohort, the initiation of dotinurad was associated with a more favorable change in the eGFR slope compared with febuxostat. However, considering the observational nature and the small sample size, these findings should be regarded as hypothesis-generating. Further large-scale studies are warranted to confirm these results.

**Supplementary Information:**

The online version contains supplementary material available at 10.1186/s12882-026-04937-7.

## Introduction

Many epidemiological studies have shown that hyperuricemia is closely associated with the development and progression of chronic kidney disease (CKD) [1–3]. The proposed pathophysiological mechanisms for uric acid–mediated kidney injury include endothelial dysfunction, inflammatory responses, and activation of the renin–angiotensin system [4, 5]. These observations have raised the question of whether correcting hyperuricemia might slow CKD progression.

However, several recent large randomized controlled trials (RCTs) have challenged this hypothesis. Trials such as FEATHER [6], CKD-FIX [7], and PERL [8] failed to demonstrate any renoprotective benefit from serum urate-lowering therapy, as none showed a significant slowing of eGFR decline with xanthine oxidase inhibitors (XOIs) treatment. Reflecting these results, the KDIGO 2024 Clinical Practice Guideline no longer recommends urate-lowering therapy for asymptomatic hyperuricemia solely for kidney protection [9].

In this context, selective urate reabsorption inhibitors (SURIs) have emerged as one of the therapeutic classes with different mechanisms of action [10]. SURI agents target urate transporter 1 (URAT1) in the proximal tubule, lowering serum uric acid by increasing renal urate excretion.

Interestingly, approximately 90% of Japanese patients with hyperuricemia are reported to have impaired urate excretion [11], and several studies have indicated an association between low uric acid excretion rates and poor renal prognosis [12, 13]. Thus, correcting hyperuricemia via increased urate excretion is a pathophysiologically rational strategy for preventing the progression of CKD. However, it remains uncertain whether increasing urate excretion provides renal benefits distinct from those achieved with XOIs.

Consequently, the negative results of RCTs using XOIs have redirected the clinical question from whether uric acid should be lowered to how it should be lowered. As SURIs act directly on the proximal tubule, a key player in CKD progression, they may exert renal effects distinct from those of XOIs. Dotinurad, a recently developed SURI, has shown evidence of short-term renal benefits [14, 15]. However, long-term comparative data between SURI and XOI therapies remain limited. Therefore, the purpose of this study was to compare changes in the eGFR slope associated with the initiation of dotinurad versus febuxostat in CKD patients with hyperuricemia due to noninflammatory etiologies, primarily diabetic kidney disease and hypertensive nephrosclerosis.

## Methods

### Study design and patients

This was a single-center retrospective cohort study conducted at Toho University Ohashi Medical Center (Departments of Cardiology, Metabolism/Endocrinology, and Neurosurgery). Patients aged ≥ 18 years with CKD and hyperuricemia who were newly started on either dotinurad or febuxostat between January 2020 and October 2023 were included. Since this was a retrospective study, drug selection was based on the attending physician’s clinical judgment. Generally, dotinurad was considered for patients suspected of having reduced uric acid excretion, while febuxostat was chosen for standard management. Hyperuricemia was defined clinically by the treating physicians (generally serum uric acid > 7.0 mg/dL or a history of gout). In this study, urate-lowering therapy was initiated in accordance with Japanese clinical practice and guidelines [16], which recommend considering intervention based on individual risk assessments. This approach differs from the KDIGO 2024 Clinical Practice Guideline, which suggests not initiating therapy solely for kidney protection in patients with asymptomatic hyperuricemia [9]. To be eligible for the analysis, patients were required to have at least one eGFR measurement within 12 months before and after therapy initiation, in addition to the baseline value.

### Exclusion criteria and CKD etiology

The major exclusion criteria were as follows: (1) CKD primarily due to specific kidney diseases such as active glomerulonephritis or polycystic kidney disease; (2) baseline eGFR < 30 mL/min/1.73 m² or any history of dialysis or kidney transplantation; (3) active malignancy; (4) any episode of acute kidney injury within 12 months before baseline; (5) concomitant use of both dotinurad and febuxostat; (6) switch from other XOIs within 6 months. The primary cause of CKD for each patient was determined from the physician’s clinical diagnosis in the medical records. Diagnoses of diabetic kidney disease or hypertensive nephrosclerosis were based on clinical history (e.g., long-standing diabetes with diabetic retinopathy or long-term hypertension without significant proteinuria or hematuria). Patients with active glomerulonephritis, polycystic kidney disease, or other specific nephropathies were excluded on the basis of specialist evaluations or prior kidney biopsy results.

### Data collection and variables

Background characteristics, laboratory data, and medication information were extracted from electronic medical records. Data were collected at baseline therapy initiation, defined as 0 months [0 M], and at predefined time points: − 12 M, − 6 M, and − 3 M before initiation and + 3 M, + 6 M, and + 12 M after initiation. For each time point, the value closest to the target date (within ± 1 month) was used. Baseline covariates included age, sex, body mass index (BMI), eGFR, serum uric acid, comorbid conditions such as hypertension, diabetes mellitus, history of heart disease, history of cerebrovascular disease, and major concurrent medications such as renin–angiotensin system (RAS) inhibitors, sodium–glucose cotransporter-2 (SGLT2) inhibitors, and loop diuretics.

### Outcome measures

The primary outcome was the change in the annual eGFR slope (mL/min/1.73 m² per year) before and after therapy initiation. Specifically, we compared each patient’s eGFR slope during the 12 months prior to initiation (–12 M to 0 M) with the slope during the 12 months after initiation (0 M to + 12 M). The eGFR was calculated via the revised Japanese equation, which is based on the serum creatinine level, as recommended by the Japanese Society of Nephrology [17].

The secondary outcomes were as follows: (1) among patients with a baseline serum uric acid concentration ≥ 7.0 mg/dL, the proportion achieving a serum uric acid concentration < 6.0 mg/dL at 12 months after initiation; and (2) the proportion of patients who experienced a ≥ 30% decline in the eGFR from the baseline value at any point during the 12-month follow-up.

### Statistical analysis

Baseline patient characteristics are summarized as the means ± standard deviations (SDs) for approximately normally distributed continuous variables or medians [interquartile ranges] for variables with skewed distributions. Normality was assessed with the Shapiro–Wilk test. For between-group comparisons at baseline, we used Student’s *t*-test for normally distributed continuous variables or the Wilcoxon rank-sum test for nonparametric continuous variables and Fisher’s exact test for categorical variables. To quantify the baseline imbalance between groups, we calculated the absolute standardized mean difference (SMD) for each variable.

The linearity assumption was verified by visual inspection of individual eGFR trajectories using spaghetti plots, which confirmed the absence of systematic non-linear patterns. For the primary outcome analysis, we employed a linear mixed-effects model (LMM) to make use of all available longitudinal eGFR data and account for within-patient correlations [18, 19]. The model included eGFR as the dependent variable and fixed effects for time (continuous, measured in months from baseline), treatment group (dotinurad vs. febuxostat), period (pre-treatment vs. post-treatment), and all interaction terms. In particular, the model included a three-way interaction term (group × period × time), which allowed us to explicitly evaluate the ‘difference-in-differences’ that is, whether the change in eGFR slope from the pre-treatment to the post-treatment period differed significantly between the two groups. The model incorporated random intercepts and random slopes for each patient and was adjusted for predefined clinically important covariates: age, sex, BMI, baseline eGFR, baseline serum uric acid, hypertension, diabetes mellitus, history of heart disease, use of RAS inhibitors, SGLT2 inhibitors, and loop diuretics. To assess the robustness of our findings, a sensitivity analysis was conducted on a subcohort of patients with complete eGFR data at the − 12, 0, and + 12-month time points. To examine pre- and post-treatment trends separately, we additionally evaluated the group × time interaction within each period (− 12 to 0 months and 0 to + 12 months) to assess potential differences in the baseline and post-treatment slopes. All analyses were performed using Stata/MP version 18.5 (StataCorp LLC, College Station, TX, USA). A two-sided *p* value < 0.05 was considered statistically significant. *P*-values for secondary outcomes were not adjusted for multiple comparisons and were interpreted as exploratory.

## Results

### Patient selection and baseline characteristics

Out of 165 patients assessed for eligibility, 84 were excluded on the basis of the prespecified criteria (see Supplemental Fig. 1 for details of exclusions). The remaining 81 patients (31 on dotinurad and 50 on febuxostat) were included in the analysis.

As shown in Table 1, the baseline characteristics were generally similar between the two groups, with a few exceptions. The dotinurad group had a higher median baseline eGFR than the febuxostat group did. The median baseline serum uric acid level was significantly lower in the dotinurad group. A history of gout was observed in only one patient in each group. Other background factors, including age, sex, BMI, comorbidities (hypertension, diabetes, cardiovascular disease), and the use of RAS inhibitors, SGLT2 inhibitors, or diuretics, were comparable between the groups (all *p* > 0.1, with low SMD values). Given the baseline differences in eGFR and serum uric acid, these variables were adjusted for in the multivariable analysis.

**Table 1:** Baseline characteristics of patients treated with dotinurad or febuxostat

Characteristic	All patients (*n* = 81)	Febuxostat (*n* = 50)	Dotinurad (*n* = 31)	*p*-value	SMD (absolute)
Age (years)	70.3 ± 12.1	71.6 ± 12.2	68.1 ± 11.8	0.212	0.289
Male sex (%)	65 (80.2%)	41 (82.0%)	24 (77.4%)	0.775	0.114
BMI (kg/m²)	24.3 ± 3.4	24.1 ± 3.4	24.4 ± 3.3	0.698	0.090
Baseline eGFR (mL/min/1.73 m²)	46.8 [39.7–53.1]	44.1 [36.0–49.9]	50.9 [42.4–55.9]	0.010	0.625
Baseline UA (mg/dL)	8.6 [7.7– 9.1]	9.0 [8.1– 9.2]	7.8 [7.2– 8.9]	0.007	0.578
History of gout (%)	2 (2.5%)	1 (2.0%)	1 (3.2%)	1.000	0.075
History of heart disease (%)	47 (58.0%)	30 (60.0%)	17 (54.8%)	0.652	0.104
History of brain disease (%)	11 (13.6%)	8 (16.0%)	3 (9.7%)	0.518	0.189
Hypertension (%)	61 (75.3%)	41 (82.0%)	20 (64.5%)	0.111	0.395
Diabetes mellitus (%)	27 (33.3%)	16 (32.0%)	11 (35.5%)	0.811	0.074
Hyperlipidemia (%)	51 (63.7%)	33 (67.3%)	18 (58.1%)	0.477	0.192
Proteinuria* (%)	18 (31.6%)	10 (31.3%)	8 (32%)	1.000	0.016
RASi (%)	55 (67.9%)	35 (70.0%)	20 (64.5%)	0.632	0.117
SGLT2i (%)	13 (16.0%)	8 (16.0%)	5 (16.1%)	1.000	0.004
Loop diuretic (%)	23 (28.4%)	17 (34.0%)	6 (19.4%)	0.207	0.331

Data are presented as mean ± SD, median [IQR], or n (%)

Abbreviations: BMI, body mass index; UA, uric acid; eGFR, estimated glomerular filtration rate; history of heart disease, heart failure, myocardial infarction, or cardiomyopathy; history of brain disease, history of ischemic stroke or intracranial hemorrhage; RASi, renin–angiotensin system inhibitor (including both angiotensin II receptor blocker [ARB] and angiotensin-converting enzyme inhibitor [ACEI]); SGLT2i, sodium–glucose cotransporter 2 inhibitor; SMD, standardized mean difference (absolute)

* Proteinuria was defined as a dipstick urinalysis result of ≥ 1+ (≥ 30 mg/dL of urinary protein) and was assessed in patients with available urinalysis data (*n* = 57)

### Changes in concomitant medications

We reviewed changes in RAS inhibitors and SGLT2 inhibitors during the 2-year observation period. Adjustments to RAS inhibitors (including initiation, discontinuation, or dose change) were observed in 21 patients (25.9%) in total, with no significant difference between the dotinurad (22.6%) and febuxostat (28.0%) groups (*p* = 0.80). Similarly, changes in SGLT2 inhibitors occurred in 7 patients (8.6%) overall, with comparable frequencies between the dotinurad (9.7%) and febuxostat (8.0%) groups (*p* > 0.99). Because these records included changes that occurred before the index date, we additionally summarized post-index changes (months ≥ 0) to better contextualize potential confounding of the post-treatment eGFR trajectory. Post-treatment changes in RAS inhibitors occurred in 12 patients (median timing: 3.7 months [IQR 2.6–7.0, range 1.6–10.2]). Notably, the majority of these adjustments occurred in the febuxostat group (*n* = 10) rather than the dotinurad group (*n* = 2). Changes in SGLT2 inhibitors occurred in 3 patients (median timing: 7.9 months [IQR 7.1–10.4, range 7.1–10.4]).

### Pre- and post-treatment eGFR slopes

During the pre-treatment period (− 12 to 0 months), the annual eGFR slope was steeper in the dotinurad group (− 2.8 mL/min/1.73 m² per year) than in the febuxostat group (− 0.3 mL/min/1.73 m² per year). The group × time interaction within the pre-treatment period was not statistically significant (*p* = 0.240), indicating that the pre-treatment trends were not significantly different between the groups. During the post-treatment period (0 to + 12 months), the slopes diverged, with + 1.4 mL/min/1.73 m² per year in the dotinurad group and − 0.8 mL/min/1.73 m² per year in the febuxostat group. These descriptive findings suggested that the improvement in the eGFR trajectory after dotinurad initiation was greater than that after febuxostat.

### Primary outcome

**Fig. 1:**
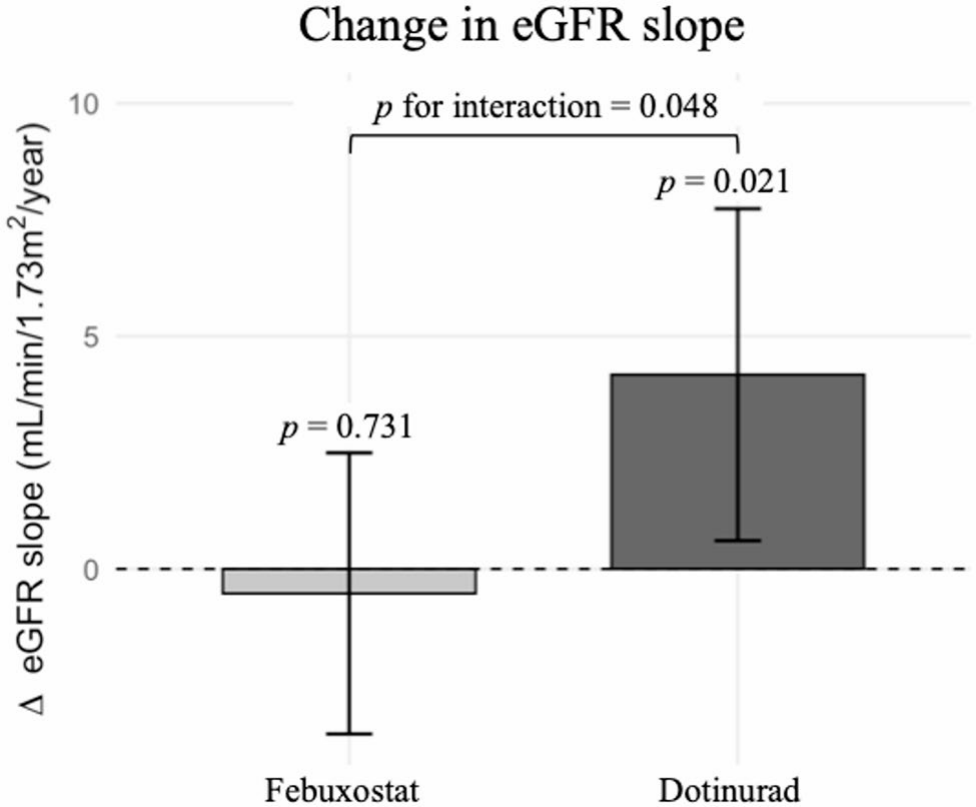
Short-term changes in the annual eGFR slope before and after treatment (model-estimated slopes with 95% CIs). The bars show the change in the annual eGFR slope within each group (difference between the pre-treatment [–12 to 0 months] and post-treatment [0 to + 12 months] periods). The error bars represent the 95% confidence intervals. *P* values above each bar indicate the significance of the within-group slope change (Wald test), and the value at the top indicates the *p*-value for the interaction (difference-in-differences) between the two groups (from the adjusted LMM). The model included a random intercept and slope for each patient and was adjusted for age, sex, BMI, baseline eGFR, baseline uric acid, hypertension, diabetes mellitus, history of heart disease, RAS inhibitor use, SGLT2 inhibitor use, and loop diuretic use

After multivariable adjustment, the LMM analysis revealed a statistically significant difference in the change in the eGFR slope from before to after the intervention between the two groups (interaction term for group × period × time, *p* = 0.048). In the dotinurad group, the model-estimated annual eGFR slope changed from − 2.8 mL/min/1.73 m² per year (95% CI − 6.0 to + 0.4) during the year before treatment to + 1.4 mL/min/1.73 m² per year (95% CI − 1.0 to + 3.7) in the year after starting treatment. This within-group change (+ 4.2 mL/min/1.73 m² per year) was statistically significant (*p* = 0.021). In contrast, the febuxostat group’s eGFR slope changed only slightly, from − 0.3 (95% CI − 3.0 to + 2.5) to − 0.8 (95% CI − 2.6 to + 1.0) mL/min/1.73 m² per year after treatment, a small and non-significant change (within-group difference − 0.5 mL/min/1.73 m² per year, *p* = 0.731). Accordingly, only dotinurad was associated with a statistically significant stabilization of the eGFR trajectory over one year, whereas febuxostat was not. This divergence in eGFR trajectories underpinned the significant between-group difference in the primary outcome (Fig. 1).

### Secondary outcomes

Among patients with a baseline serum uric acid concentration ≥ 7.0 mg/dL, the rate of achieving the target uric acid level < 6.0 mg/dL at 12 months did not differ significantly between the dotinurad and febuxostat groups (Table 2). Although the febuxostat group had a greater average baseline serum uric acid level and a slightly greater proportion of patients who reached the uric acid target, this difference was not statistically significant. In terms of absolute values at 12 months, we evaluated both median and mean values to ensure consistency with baseline characteristics and the linear mixed-effects model analysis. In the febuxostat group, the median eGFR changed from 44.1 [36.0–49.9] at baseline to 43.9 [35.4–51.9] mL/min/1.73 m² at 12 months (mean: 44.1 ± 8.2 to 43.4 ± 9.8 mL/min/1.73 m²). The median serum uric acid levels decreased from 9.0 [8.1–9.2] to 5.5 [5.1–6.6] mg/dL (mean: 9.0 ± 1.2 to 5.8 ± 1.3 mg/dL). In the dotinurad group, the median eGFR changed from 50.9 [42.4–55.9] at baseline to 49.5 [46.3–55.6] mL/min/1.73 m² at 12 months (mean: 49.2 ± 8.1 to 50.1 ± 10.1 mL/min/1.73 m²). The median serum uric acid levels decreased from 7.8 [7.2–8.9] to 6.2 [5.5–6.9] mg/dL (mean: 8.1 ± 1.5 to 6.1 ± 1.2 mg/dL).

Furthermore, the proportion of patients who experienced a ≥ 30% decline in eGFR from baseline at any time during follow-up was low in both groups, and there was no significant difference between the groups at this endpoint (only a few patients in each group experienced such a decline, with similar event rates of < 10% in both groups). 

**Table 2:** Uric acid target attainment by 12 months among patients with baseline uric acid ≥ 7.0 mg/dL. The target was defined as a serum uric acid concentration < 6.0 mg/dL at 12 months. Between-group differences were evaluated via Fisher’s exact test

	Febuxostat (n = 48)	Dotinurad (n = 26)	p-value
Achieved UA <6.0 at 12M, n (%)	26 (54.2%)	10 (38.5%)	0.230

### Changes in proteinuria (exploratory analysis)

Paired baseline and 12-month dipstick data were available for 48 patients (dotinurad *n* = 21, febuxostat *n* = 27). In the dotinurad group, grades remained unchanged in 16 (76.2%), improved in 1 (4.8%), and worsened in 4 (19.0%). In the febuxostat group, grades were unchanged in 20 (74.1%), improved in 4 (14.8%), and worsened in 3 (11.1%). No significant longitudinal changes were observed in either group (Wilcoxon signed-rank test, dotinurad *p* = 0.375 and febuxostat *p* = 0.766). Additionally, the proportion of patients with positive proteinuria (≥ 1+) did not change significantly (McNemar’s test, dotinurad *p* = 0.180 and febuxostat *p* = 0.564).

## Discussion

In this real-world CKD population, initiation of dotinurad was associated with a more stable eGFR slope than febuxostat initiation. This change was characterized by the stabilization of a previously declining eGFR trajectory in the dotinurad group, whereas no similar change was observed with febuxostat. A key strength of this investigation is its longitudinal design, which characterizes the evolution of renal function over time rather than relying on simple two-point comparisons that may fail to capture underlying trends.

Our 12-month observation period extends previous reports of stabilization of the eGFR with dotinurad, which were limited to shorter follow-up periods (3–6 months) [14, 20]. These results support the possibility that the stabilization of renal function with SURI therapy can be sustained over a longer term. Moreover, considering that the major RCTs using XOIs did not demonstrate renoprotection [6–8], the finding that a mechanistically different urate-lowering agent showed a beneficial association is clinically noteworthy and hypothesis-generating, although not conclusive.

To contextualize our findings, we summarized recent studies evaluating the renoprotective potential of dotinurad in Supplemental Table 2. Consistent with our results, Amano et al. [14] and Takata et al. [21] reported improved eGFR changes compared with pre-treatment periods or with febuxostat treatment. However, both studies were limited by relatively short observation periods of approximately 3 months. The present study extends these observations by suggesting that stabilization of the eGFR slope may be sustained over a longer duration of 12 months. In contrast, Motomura et al. [22] found no significant improvement in the eGFR slope among patients with advanced CKD, suggesting that the renoprotective potential of dotinurad may depend on residual kidney function. Similarly, Kurihara et al. [23] observed improvements only in patients with severe renal impairment, although differences in study design and sample characteristics may explain the apparent discrepancies between reports. The relatively pronounced effect size in our cohort may reflect the fact that most participants had moderate CKD, in whom sufficient functional nephrons may remain to benefit from URAT1 inhibition. Furthermore, the application of a difference-in-differences analytical approach allowed us to quantify a reversal in the eGFR trajectory from the pre-treatment to the post-treatment period.

The observed eGFR improvement does not appear to be solely attributable to urate reduction. The secondary outcomes results are illustrative in this regard: the degree of serum uric acid reduction was similar in the two groups, yet the eGFR slope changed only in the dotinurad group. This finding suggests that any renoprotective effect may depend not only on *how much* the serum uric acid level is lowered but also on *how* it is lowered. In other words, the mechanism of urate reduction could influence renal outcomes. The hypothesis that “the way uric acid is lowered can affect kidney outcomes” is supported by a recent study identifying URAT1 as a potential therapeutic target for slowing CKD progression. This concept may become an important consideration in future therapeutic strategies for hyperuricemia in CKD patients.

The biological plausibility of a mechanism-dependent effect is supported by SURI’s mode of action at the proximal tubule. Excess reabsorbed uric acid within proximal tubular cells has direct cytotoxic effects and can induce tubular apoptosis via NOX4-dependent oxidative stress [24]. Additionally, uric acid has been shown to trigger inflammatory responses in kidney tubules through the LDHA/ROS/NLRP3 inflammasome pathway [25]. Thus, uric acid is implicated in kidney injury through multiple intracellular routes. By inhibiting URAT1 and reducing intracellular urate accumulation, SURI agents may directly attenuate these injurious processes and thereby exert tubule-protective effects.

Notably, sodium–glucose cotransporter-2 (SGLT2) inhibitors, which act on the proximal tubule, confer renoprotective benefits while also promoting uric acid excretion. The uricosuric effect of SGLT2 inhibitors has been linked to the modulation of URAT1, supporting the notion that a “proximal tubule-targeted” strategy may be effective for kidney protection [26, 27]. In the future, combination therapy with a SURI such as dotinurad with an SGLT2 inhibitor may be a potential strategy worthy of further investigation for additive renoprotective effects.

From a clinical perspective, adding a SURI with a different mechanism of action could be a promising option for CKD patients who continue to experience eGFR decline despite standard care (e.g., RAS blockers and SGLT2 inhibitors). Our study population consisted of patients with common CKD etiologies and deliberately excluded special conditions such as active glomerulonephritis, which enhances the applicability of our findings to typical CKD practice. Nonetheless, further investigations are needed to explore the potential heterogeneity of the treatment effect across different underlying kidney diseases and to examine any interactions with existing therapies.

It is noteworthy that the dotinurad group exhibited a steeper decline in eGFR during the pre-treatment period compared to the febuxostat group. This difference likely reflects indication bias inherent in real-world clinical practice, whereby dotinurad may have been preferentially prescribed to patients with more rapidly declining renal function or perceived treatment difficulty. Such selection of patients with extreme pre-treatment trajectories inherently introduces the potential for regression to the mean (RTM). We acknowledge that RTM may have contributed substantially to the magnitude of the observed change in the dotinurad group, given its steeper pre-treatment decline. However, the transition from a declining to a positive eGFR slope may not be solely attributable to natural variation, and a pharmacological effect of dotinurad cannot be excluded. Nevertheless, our findings should be interpreted as associative rather than evidence of a causal treatment effect. This study has several limitations. First, the small sample size, particularly in the dotinurad group, limited the statistical power of sensitivity analyses, including IPTW, and increases the possibility that the observed associations are fragile. Although detailed results are provided in Supplemental Table 1, residual and unmeasured confounding at baseline cannot be fully excluded in this observational study. Second, specific markers of tubular injury (e.g., L-FABP, NAG, or KIM-1) were not measured owing to the retrospective nature of the study; therefore, the proposed renoprotective mechanism remains speculative. Future prospective studies including these biomarkers are needed to clarify the precise mechanism of renoprotection. Third, our analysis focused on the one-year eGFR slope as a surrogate outcome; the impact on harder endpoints such as progression to end-stage renal disease, cardiovascular events, or mortality remains unknown. Fourth, the retrospective nature of the study led to variability in the timing of eGFR measurements and in medication adjustments. Although these changes were documented, residual confounding related to the clinical rationale behind these adjustments cannot be entirely excluded. Fifth, no significant longitudinal changes in proteinuria were observed in either group, which may be due to the relatively short observation period and the qualitative nature of the dipstick assessment. Furthermore, the available data were incomplete and lacked quantitative measures, which precluded stratified analyses by albuminuria.

## Conclusion

In conclusion, the initiation of dotinurad was associated with a more favorable change in the eGFR slope compared with febuxostat in CKD patients with hyperuricemia. Patients in the dotinurad group were observed to have, on average, a relative change from a declining to a stable or improving eGFR trajectory over one year, whereas those in the febuxostat group did not show such improvement. However, considering the observational nature and the small sample size, these findings should be regarded as hypothesis-generating. Further large-scale, prospective studies are warranted to determine whether this association represents a causal relationship and translates into meaningful clinical benefits for this population.

## Supplementary Information

Below is the link to the electronic supplementary material.

Supplementary Material 1

Supplementary Material 2

## Data Availability

The datasets generated and/or analyzed during the current study are available from the corresponding author upon reasonable request.
