# Supplementary material for: Effect of dotinurad versus febuxostat on the one-year eGFR slope in CKD patients with hyperuricemia: a retrospective cohort study
Source: BMC Nephrol. 2026 Apr 10;27:322. doi: 10.1186/s12882-026-04937-7 (PMC13192177; doi:10.1186/s12882-026-04937-7)
Supplement: Supplementary file 1 — Supplementary Material 1 [file 12882_2026_4937_MOESM1_ESM.docx]

Supplement （Additional files）

Flow diagram of participant selection. Among the 165 patients initially assessed for eligibility, 56 were excluded because of insufficient follow-up data, 21 because of switching from other xanthine oxidase inhibitors to febuxostat, 4 because of prior acute kidney injury, 2 because of concomitant use of dotinurad and febuxostat, and 1 because of active nephritis. A total of 81 patients were included in the final analysis （see *Supplemental Figure 1*.）.

*Supplemental Figure 1*.

Supplemental Table 1. Results of sensitivity analysis using Inverse Probability of Treatment Weighting (IPTW).

| Outcome | Estimate  (Difference-in-Differences) | 95% Confidence Interval | *p*-value |
| --- | --- | --- | --- |
| Annual eGFR slope (mL/min/1.73 m²/year) | +10.41 | -1.08 to +21.90 | 0.076 |

Note: The analysis was performed using a linear mixed-effects model weighted by the inverse probability of treatment weighting (IPTW) to estimate the Average Treatment Effect on the Treated (ATT). The model was adjusted for baseline eGFR, baseline serum uric acid, age, sex, BMI, hypertension, diabetes mellitus, history of heart disease, and use of RAS inhibitors, SGLT2 inhibitors, and loop diuretics. Abbreviations: ATT, average treatment effect on the treated; CI, confidence interval; eGFR, estimated glomerular filtration rate.

Supplemental Text: Complete-pair sensitivity analysis

As a sensitivity analysis, we restricted the cohort to patients with complete eGFR measurements at −12, 0, and +12 months (“complete-pair” cohort). The analysis replicated the primary model specification: a linear mixed-effects model with a random intercept and random slope for time (years) at the patient level and unstructured covariance. Fixed effects included treatment group (dotinurad vs. febuxostat), period (pre- vs. post initiation), continuous time (years), and their three-way interaction (group × period × time), adjusted for baseline eGFR and serum uric acid, and the prespecified covariates age, sex, hypertension, diabetes, use of renin–angiotensin system inhibitors, use of SGLT2 inhibitors, history of heart disease, loop diuretic use, and BMI. Annual eGFR slopes for each treatment group and period were estimated via marginal effects from the mixed-effects model. Within-group post–pre differences in slopes were evaluated via linear contrasts, and the between-group difference-in-differences (DiD) in slope was assessed via the Wald test.

Supplemental Results: Complete-pair sensitivity analysis

Among the 81 eligible patients, 56 had complete eGFR data at −12, 0, and +12 months (febuxostat n = 32; dotinurad n = 24). In this subset, the within-group post–pre change in annual eGFR slope was −1.64 (95% CI −4.74 to 1.47) mL/min/1.73 m² per year for febuxostat (*p* = 0.301) and +4.48 (95% CI 0.88 to 8.08) mL/min/1.73 m² per year for dotinurad (*p* = 0.015). The resulting difference-in-differences was 6.11 mL/min/1.73 m² per year (Wald *p* = 0.012), which is consistent with the primary analysis and indicates a robust association favoring dotinurad when it is restricted to patients with complete measurements.
