# Supplementary material for: Effect of dotinurad versus febuxostat on the one-year eGFR slope in CKD patients with hyperuricemia: a retrospective cohort study
Source: BMC Nephrol. 2026 Apr 10;27:322. doi: 10.1186/s12882-026-04937-7 (PMC13192177; doi:10.1186/s12882-026-04937-7)
Supplement: Supplementary file 2 — Supplementary Material 2 [file 12882_2026_4937_MOESM2_ESM.docx]

Supplemental Table 2. Summary of recent studies evaluating the renoprotective effects of dotinurad in patients with CKD.

| Study (First Author, Year) | Study Design | Sample Size (n) | Baseline eGFR (mL/min/1.73 m²) | Observation Period | Comparator | Key Renal Outcome |
| --- | --- | --- | --- | --- | --- | --- |
| Present study | Retrospective Cohort | Dotinurad (n=31), Febuxostat (n=50) | 50.9 (Dotinurad),  44.1 (Febuxostat) | 12 months | Febuxostat | Dotinurad significantly improved eGFR slope vs. Febuxostat (Interaction p=0.048). Dotinurad: -2.8 to +1.4 mL/min/1.73 m²/year. |
| Amano et al. [14] (2024) | Retrospective (Single-arm) | Dotinurad (n=35) | 31.8 ± 16.4 | 3 months | None (Pre-treatment period) | Significant improvement in eGFR change.Pre: -3.7 ± 5.6 → Post: +4.7 ± 9.5 mL/min/1.73 m² (over 3 months). |
| Takata et al.  [21] (2025) | Retrospective Cohort | Dotinurad (n=29), Febuxostat (n=29) | 33.9 ± 15.2 (Dotinurad), 33.4 ± 19.6 (Febuxostat) | 3 months | Febuxostat | % change in eGFR was significantly higher in Dotinurad group (+7.45%) compared to Febuxostat group (-0.42%) (p=0.042). |
| Motomura et al. [22] (2025) | Retrospective (Single-arm) | Dotinurad (n=14) | 24.9 [18.3–35.5] (median) | 6 months | None (Pre-treatment period) | No significant change in eGFR slope (p=0.72). Significant reduction in urinary protein-to-creatinine ratio (p<0.05). |
| Kurihara et al. [23] (2024) | Retrospective (Single-arm) | Dotinurad (n=53) | 38.7 ± 17.0 | 9.8 ± 4.5 months (mean) | None | No significant change in overall cohort.Significant eGFR improvement observed only in severe CKD group (eGFR <30, p=0.032). |

Abbreviations: CKD, chronic kidney disease; eGFR, estimated glomerular filtration rate. Note: Baseline eGFR values are presented as mean ± standard deviation or median [interquartile range]. Comparisons refer to either between-group differences (vs. comparator) or within-group changes (vs. pre-treatment period), as specified in the outcome column.

[14] Amano H, Kobayashi S, Terawaki H. Dotinurad restores exacerbated kidney dysfunction in hyperuricemic patients with chronic kidney disease. BMC Nephrol 2024;25:97.

[21] Takata T, Taniguchi S, Mae Y, Kageyama K, Fujino Y, Iyama T, et al. Comparative assessment of the effects of dotinurad and febuxostat on the renal function in chronic kidney disease patients with hyperuricemia. Sci Rep 2025;15:8990.

[22] Motomura T, Higashi M, Hattori A, Akiyama R, Kai H. Efficacy and Safety of Dotinurad in Patients with Advanced Chronic Kidney Disease. Intern Med 2025.

[23] Kurihara O, Yamada T, Kato K, Miyauchi Y. Efficacy of dotinurad in patients with severe renal dysfunction. Clin Exp Nephrol 2024;28:208–216.
